# Prediction of Patients with Acute Cholecystitis Requiring Emergent Cholecystectomy: A Simple Score

**DOI:** 10.1155/2010/901739

**Published:** 2010-06-08

**Authors:** Wael N. Yacoub, Mikael Petrosyan, Indu Sehgal, Yanling Ma, Parakrama Chandrasoma, Rodney J. Mason

**Affiliations:** ^1^Department of Surgery, Keck School of Medicine, University of Southern California, Los Angeles, CA 90089, USA; ^2^Department of Pathology, Keck School of Medicine, University of Southern California, Los Angeles, CA 90089, USA; ^3^Department of Surgery, LAC+USC Medical Center, 1200 North State Street, no. 10850, Los Angeles, CA 90033, USA

## Abstract

The objective was to develop a score, to stratify patients with acute cholecystitis into high, intermediate, or low probability of gangrenous cholecystitis. The probability of gangrenous cholecystitis (score) was derived from a logistic regression of a clinical and pathological review of 245 patients undergoing urgent cholecystectomy. Sixty-eight patients had gangrenous inflammation, 132 acute, and 45 no inflammation. The score comprised of: age > 45 years (1 point), heart rate > 90 beats/min (1 point), male (2 points), Leucocytosis > 13,000/mm^3^ (1.5 points), and ultrasound gallbladder wall thickness > 4.5 mm (1 point). The prevalence of gangrenous cholecystitis was 13% in the low-probability (0–2 points), 33% in the intermediate-probability (2–4.5 points), and 87% in the high probability category (>4.5 points). A cutoff score of 2 identified 31 (69%) patients with no acute inflammation (PPV 90%). This scoring system can prioritize patients for emergent cholecystectomy based on their expected pathology.

## 1. Introduction

Surgeons working in large medical centers frequently encounter patients with a suspected diagnosis of acute cholecystitis. Early identification of patients with advanced cholecystitis would aid the surgeon in prioritizing patients for operation, especially when multiple patients present with similar diagnoses. This is not an uncommon occurrence in busy medical centers that have limited operating room availability due to several surgical services competing for operating room time. 

 Accurately predicting which patients will have advanced pathologic inflammation of the gallbladder (i.e., gangrenous cholecystitis) would be of great value to the busy surgeon to prioritize those patients for early operation and prevent progression to perforation. Previous diagnostic models have only identified risk factors for gangrenous cholecystitis such as male gender, older age, white blood cell count elevation, and diabetes [1–3]. However, a simple predictive scoring system as exists for appendicitis [4] or pancreatitis [5] does not exist for cholecystitis, a very common surgical disease. 

 We attempted to identify preoperative clinical predictors of pathologic gangrenous inflammation of the gallbladder and use these to develop a simple predictive clinical score. This score would then be used to prioritize patients for operations as indicated.

## 2. Methods

Patients who present to the LAC+USC Medical Center with a suggested diagnosis of acute calculous cholecystitis are evaluated by the Emergency Surgery Service. If a clinical diagnosis of acute cholecystitis is made patients are admitted for intravenous hydration, antibiotics, and booked for operation on the next available operating room time. In October 2004 a large database was started to include demographic, clinical, laboratory, radiographic, and pathologic information of all patients seen by the Emergency Surgery Service. This prospectively collected data was then queried for patients treated for acute cholecystitis from October 2004 to February 2007.

Patients treated for suspected acute cholecystitis with cholecystectomy (open or laparoscopic) within 10 days of onset of their symptoms and who had complete data available were included for further analysis. Patients with alternate diagnoses of choledocholithiasis or gallstone pancreatitis were excluded.

A blinded expert pathologist then performed a comprehensive pathology slide review on all patients that met the search criteria. The pathologist was blinded to all clinical or original pathologic information on the patients. This was done to objectify the pathologic diagnosis of cholecystitis and to remove any bias on the part of the pathologist.

 Pathologic diagnoses were made using current standard definitions and criteria used in surgical pathology literature, with similar definitions described by Fitzgibbons et al. in their review [4]. Possible pathologic diagnoses included (1) chronic cholecystitis, characterized by wall thickening by fibrosis and presence of chronic inflammatory cells only without neutrophils, (2) acute on chronic cholecystitis, characterized by superimposition of neutrophilic infiltration on chronic cholecystitis, (3) acute cholecystitis, characterized by acute inflammation without significant fibrous thickening of gall bladder wall, or (4) gangrenous cholecystitis, where the acute inflammation was associated with evidence of necrosis of the gall bladder wall.

 Collected data included demographic characteristics, clinical signs and symptoms suggestive of acute cholecystitis, and sonographic findings. All variable included in the analysis are listed in Table 1. 

### 2.1. Statistical Analysis

We performed univariate analysis to select predictors with statistically significant association with gangrenous cholecystitis. We assessed significance by using the chi-square test for nominal categorical variables and the Mann-Whitney *U* test for continuous variables. A 2-tailed *P* value less than  .05 indicated statistical significance. Variables with statistical significance were then used in a multivariate logistic regression model. Nonstatistically significant variables were then removed and a regression coefficient was calculated for each statistically significant variable in the final model. We assigned points for the score according to the regression coefficient (i.e., strength of association), with 1 point corresponding to a value close to the smallest regression coefficient and serving as the least common denominator for assigning point values for the other score items.

### 2.2. Score Derivation

We then computed the score for each patient, performed a receiver operating characteristic (ROC) curve analysis, and computed the area under the ROC curve and its corresponding 95% CI. Finally, we chose the cutoff value that discriminated among the low-, intermediate-, and high-probability groups to identify (1) a low-probability group with a prevalence of gangrenous cholecystitis of approximately 10% and (2) a high-probability group with a prevalence of gangrenous cholecystitis more than 60%. We assessed the predictive accuracy of the final score categories (i.e., positive predictive value) by the proportion of patients with gangrenous cholecystitis in each group.

### 2.3. Validation Population

The scoring system was then validated on a second population of patients admitted from March 2007 until July 2009. 

 The Institutional Review Board at the Keck School of Medicine at the University of Southern California approved the study. 

## 3. Results

Of 829 patients that presented with right upper quadrant or epigastric pain and evaluated by the Emergency Surgery Service during the study period, 245 patients met the inclusion criteria and had complete data available. The average age was 39.7 ± 12.0 years. There were 69 (28.1%) males with an average age of 41.9 years, and 176 females with an average age 38.8 years. The average time from onset of symptoms to surgery was 3.6 ± 2.4 days, and the average time from admission to surgery was 1.4 ± 1.6 days.

 The original pathology and the blinded pathology review results are presented in Table 2. Three patients were found to have xanthogranulomatous cholecystitis on blinded review, and were included in the gangrenous group for analysis. Clinical information on patients based on pathologic gangrenous cholecystitis compared with those who had acute cholecystitis and chronic cholecystitis is presented in Table 3.

 Univariate analysis identified 7 variables with statistical significant association with gangrenous cholecystitis. These include age > 45 years, male gender, nausea, temperature > 37.5 degrees Celsius, heart rate > 90 beats per minute, white blood cell count > 13,000 cells/mm^3^, and gallbladder wall thickness > 4.5 mm on ultrasound. We included all 7 variables in a multivariate logistic regression model. In the multivariate analysis, nausea and temperature were not independently associated with gangrenous cholecystitis. 

Regression coefficients were then calculated using the logistic regression model, and according to the regression coefficient points were assigned to each of the 5 variables independently associated with gangrenous cholecystitis on multivariate analysis (see Table 4). The lowest regression coefficient (0.79, age > 45 years) was assigned 1 point, and the others according to their relative proportion to this lowest common denominator. Male gender had the strongest independent association with gangrenous cholecystitis and therefore was assigned a score of 2. This resulted in a total possible score of 6.5.

**Figure 1:**
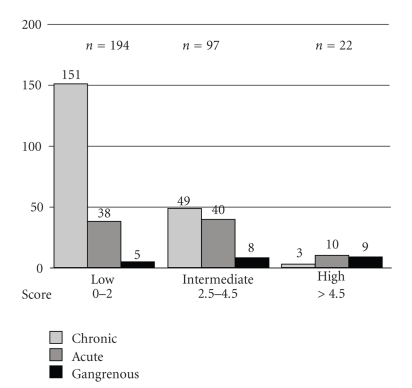
Distribution of cholecystitis according to probability group in the derivation population.

A total score was then retrospectively calculated for each of the 245 patients based on the 5 independent variables. Probability categories (low-, intermediate-, or high-probability) were then divided using cutoffs to create the low or high incidence of gangrenous cholecystitis in each category. A cutoff score of 2 was used for the low-probability group, and >4.5 for the high-probability group. Figure 1 shows the distribution of each pathologic diagnosis according to the probability category (0–2 = low, 2.5–4.5 = intermediate, or >4.5 = high). The positive predictive value of gangrenous cholecystitis in the high-probability group is 87% (13/15 patients), whereas in the low-probability group it is 13% (13/103 patients). 

The validation population consisted of 313 patients with and an average age of 39 ± 13.2 years. This included 77 (24.6%) males with an average age of 43.4 years and 236 females with an average age of 37.9 years. 

**Figure 2:**
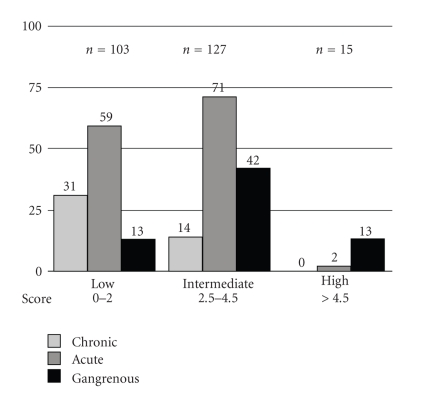
Distribution of cholecystitis according to probability group in the validation population.

 Similarly, a total score was then retrospectively calculated for each of the 313 patients in the validation population. Figure 2 shows the distribution of each pathologic diagnosis according to the probability category (0–2 = low, 2.5–4.5 = intermediate, or >4.5 = high) in the validation population. The positive predictive value of acute /gangrenous cholecystitis in the high-probability group was 86% (19/22 patients), whereas in the low-probability group the positive predictive value of gangrenous cholecystitis was 14% (5/194 patients). 

## 4. Discussion

Acute inflammation of the gallbladder is most commonly the consequence of persistent outlet obstruction of the gallbladder by gallstones. It is on the continuum of clinical and histologic findings between simple biliary colic and advanced gallbladder necrosis and perforation. The accurate clinical diagnosis of acute inflammation of the gallbladder can often be difficult due to the similarity in symptoms between an attack of biliary colic and acute cholecystitis. Biliary colic is a self-limiting, transient obstruction of the gallbladder that with repeated episodes leads chronic cholecystitis. Acute calculous cholecystitis on the other hand is an acute inflammatory process from unrelieved gallbladder obstruction that often progresses to gallbladder necrosis if left untreated (i.e., antibiotics or cholecystectomy). Early identification of patients with acute cholecystitis is critical for appropriate treatment with antibiotics and early cholecystectomy. Early cholecystectomy has been shown to be a safe treatment for patients with acute cholecystitis, and is critical in those with advanced gangrenous cholecystitis [5, 6]. Surgeons that practice in large, busy medical centers in which this disease is frequently encountered often have to deal with limited operating room resources, and hence, delaying the surgical treatment of cholecystitis. An accurate clinical tool to help in prioritizing patients with suspected cholecystitis for operative intervention would be ideal in this setting. Moreover, patients with gangrenous cholecystitis on admission need emergent surgery to prevent impending perforation.

 Previous studies have found important clinical indicators to preoperatively predict gangrenous cholecystitis [1–3]. Male gender, older age, diabetes, and elevated white blood cell count have all been found to increase the risk of having gangrene of the gallbladder. Surgeons routinely use this information along with the overall status of the patient to make clinical diagnoses and prioritize patients for the operating room. However, having a scoring system to help accurately predict the pathology of patients with suspected acute cholecystitis would be a tremendous tool for surgeons that practice in busy medical centers with limited operating room availability. Patients can be prioritized based on a clinical score to either needing emergent cholecystectomy, or cholecystectomy on a more semiurgent or possibly elective basis. 

 Attempts at accurate preoperative prediction of patients with acute cholecystitis have been made. However, only the minority of the studies reviewed by Trowbridge et al. relied on pathologic confirmation of acute cholecystitis for correlation with clinical factors [7]. It has been shown, and is likely common experience among surgeons, that the clinical and pathologic diagnoses of cholecystitis are frequently conflicting [4]. That is, patients may present with what appears to be a clinical diagnosis of acute cholecystitis, but pathologically only show chronic changes without an acute inflammatory component. It is only the acute inflammatory process due to unrelieved obstruction of the gallbladder that can progress to gallbladder necrosis if left untreated (Figure 2).

In our study we attempted to create a simple clinical score to preoperatively predict pathologic gangrenous cholecystitis. Multivariate analysis showed 5 independent variables associated with gangrenous cholecystitis: Male gender, white blood cell count > 13,000, heart rate > 90, gallbladder wall thickness > 4.5 mm, and age > 45 years. The positive predictive value of patients with a clinical score > 4.5 in having gangrenous cholecystitis on pathology nearing 90%. Nguyen et al. attempted to developed an equation based on multivariate analysis to predict gangrenous cholecystitis with a positive predictive value of 71% [3]. 

 Of interest is the finding of discrepancy between the original pathologic diagnosis and the blinded pathology slide review. This is likely due to the fact that routine pathologic examination of cholecystectomy specimens usually focuses on search for malignancy and not necessarily the accurate diagnosis of the inflammatory process. The gallbladder is already removed and accurately classifying the degree of inflammation is probably inconsequential to most pathologists. Chronic inflammation alone does not progress to necrosis, and is not an indication for emergent cholecystectomy. Studies that correlate clinical and pathologic findings ought to accurately classify the degree of inflammation on pathology in order to provide sound clinical predictors.

## 5. Conclusion

Using a simple clinical score, patients that present to the emergency room with a suspected diagnosis of acute cholecystitis can be prioritized for surgery, based on actual pathologic findings. Patients can be accurately classified into having low-, intermediate-, or high-probability of having gangrenous cholecystitis. This would aid the busy surgeon in prioritizing limited operating room resources accordingly. 

## Figures and Tables

**Table 1:** Clinical data included in univariate analysis.

	*P*-value (Gangrenous versus nongangrenous cholecystitis)
Age	<.0001
Male gender	.0001
Temperature	<.0001
Heart rate	.0054
Duration of symptoms	.1714
Nausea	.0440
Vomiting	.1280
Clinical Murphy's sign	.2390
White blood cell count	<.0001
Total bilirubin	.4322
Alkaline phosphatase	.2216
Sonographic Murphy's sign	.2483
Gallbladder wall thickness on ultrasound	.0008
Gangrenous cholecysititis on ultrasound read	.1235

**Table 2:** Pathology review results.

Pathologic Diagnosis *n* = 245	Original Pathology Report	Blinded Pathology Review
Chronic Cholecystitis	114 (47%)	45 (18%)
Acute or Acute on Chronic Cholecystitis	91 (37%)	132 (54%)
Gangrenous Cholecystitis	37 (15%)	68 (28%)

**Table 3:** Clinical characteristics of patients by pathologic diagnosis.

	Chronic Cholecystitis	Acute Cholecystitis	Gangrenous Cholecystitis	*P*-value
	*n* = 45	*n* = 132	*n* = 68	
Gender				
Male	4 (9%)	33 (25%)	32 (47%)	.0001
Female	41 (91%)	99 (75%)	36 (53%)	
Age (mean)	36.3 yrs	38.4 yrs	44.5 yrs	.0003*
Mean Admission				
Temp	98.0°F	98.4°F	99.0°F	<.0001*
Heart Rate	81	83	89	.039**
WBC (cells/mm^3^)	11,700	13,500	16,900	<.0001*
Mean GBWT on Ultrasound	3.5 mm	4.8 mm	5.6 mm	.0001^†^
Mean Time From Onset of Symptoms to Surgery	4.3 days	3.5 days	3.5 days	.011^‡^

*Gangrenous Cholecystitis versus Acute and Chronic Cholecystitis.

**Gangrenous Cholecystitis versus Acute Cholecystitis.

^†^Chronic Cholecystitis versus Gangrenous Cholecystitis and Acute Cholecystitis.

^‡^Gangrenous Cholecystitis versus Chronic Cholecystitis.

GBWT = gallbladder wall thickness.

**Table 4:** Clinical score assignment by regression coefficient.

Variable	Regression Coefficient	*P*-Value	Assigned Score
Male Gender	1.43	<.001	2
WBC > 13,000	1.03	.005	1.5
Heart Rate > 90	0.95	.007	1
GBW > 4.5 mm	0.85	.014	1
Age > 45	0.79	.019	1

Total Possible Score			6.5

## References

[1] Merriam LT, Kanaan SA, Dawes LG (1999). Gangrenous cholecystitis: analysis of risk factors and experience with laparoscopic cholecystectomy. *Surgery*.

[2] Fagan SP, Awad SS, Rahwan K (2003). Prognostic factors for the development of gangrenous cholecystitis. *American Journal of Surgery*.

[3] Nguyen L, Fagan SP, Lee TC (2004). Use of a predictive equation for diagnosis of acute gangrenous cholecystitis. *American Journal of Surgery*.

[4] Fitzgibbons RJ, Tseng A, Wang H, Ryberg A, Nguyen N, Sims KL (1996). Acute cholecystitis: does the clinical diagnosis correlate with the pathological diagnosis?. *Surgical Endoscopy*.

[5] Jarvinen HJ, Hastbacka J (1980). Early cholecystectomy for acute cholecystitis. A prospective randomized study. *Annals of Surgery*.

[6] Eldar S, Sabo E, Nash E, Abrahamson J, Matter I (1997). Laparoscopic cholecystectomy for acute cholecystitis: prospective trial. *World Journal of Surgery*.

[7] Trowbridge RL, Rutkowski NK, Shojania KG (2003). Does this patient have acute cholecystitis?. *Journal of the American Medical Association*.

